# Variable Bone Fragility Associated With an Amish *COL1A2* Variant and a Knock-in Mouse Model

**DOI:** 10.1359/jbmr.090720

**Published:** 2009-07-13

**Authors:** Ethan Daley, Elizabeth A Streeten, John D Sorkin, Natalia Kuznetsova, Sue A Shapses, Stephanie M Carleton, Alan R Shuldiner, Joan C Marini, Charlotte L Phillips, Steven A Goldstein, Sergey Leikin, Daniel J McBride

**Affiliations:** 1 Orthopaedic Research Laboratories, Department of Orthopaedic Surgery, University of Michigan Ann Arbor, MI, USA; 2 Division of Endocrinology, Diabetes & Nutrition, University of Maryland Baltimore Baltimore, MD, USA; 3 Division of Gerontology, University of Maryland Baltimore and the Baltimore VA Medical Center, Geriatric Research, Education and Clinical Center (GRECC) Baltimore, MD, USA; 4 National Institute of Child Health and Human Development, National Institutes of Health Bethesda, MD, USA; 5 Department of Nutritional Sciences, Rutgers University New Brunswick, NJ, USA; 6 Department of Biochemistry, University of Missouri–Columbia Columbia, MO, USA

**Keywords:** osteogenesis imperfecta, bone, collagen, knock-in, rodent

## Abstract

Osteogenesis imperfecta (OI) is a heritable form of bone fragility typically associated with a dominant *COL1A1* or *COL1A2* mutation. Variable phenotype for OI patients with identical collagen mutations is well established, but phenotype variability is described using the qualitative Sillence classification. Patterning a new OI mouse model on a specific collagen mutation therefore has been hindered by the absence of an appropriate kindred with extensive quantitative phenotype data. We benefited from the large sibships of the Old Order Amish (OOA) to define a wide range of OI phenotypes in 64 individuals with the identical *COL1A2* mutation. Stratification of carrier spine (L1–4) areal bone mineral density (aBMD) *Z*-scores demonstrated that 73% had moderate to severe disease (less than −2), 23% had mild disease (−1 to −2), and 4% were in the unaffected range (greater than −1). A line of knock-in mice was patterned on the OOA mutation. Bone phenotype was evaluated in four F_1_ lines of knock-in mice that each shared approximately 50% of their genetic background. Consistent with the human pedigree, these mice had reduced body mass, aBMD, and bone strength. Whole-bone fracture susceptibility was influenced by individual genomic factors that were reflected in size, shape, and possibly bone metabolic regulation. The results indicate that the *G610C OI (Amish)* knock-in mouse is a novel translational model to identify modifying genes that influence phenotype and for testing potential therapies for OI. © 2010 American Society for Bone and Mineral Research

## Introduction

Osteogenesis imperfecta (OI) is a heritable form of bone fragility with an overall spectrum of disease severity that ranges from a perinatal lethal form to mild disease that can remain clinically silent.(1–3) Multiple fractures is the principal clinical presentation that brings OI patients to medical attention. Additional phenotype traits can include bone deformity (e.g., long bone bowing), short stature, blue sclerae, dentinogenesis imperfecta (DI), and hearing loss. Sillence originally classified OI into types I to IV on the basis of clinical presentation, radiographic features, and mode of inheritance.(4,5) OI types I to IV typically are associated with heterozygous mutations in the genes (*COL1A1* and *COL1A2*) that encode the α chains of type I procollagen molecules. Currently, more than 800 *COL1A1* and *COL1A2* mutations have been identified in OI patients, with many examples of recurrent mutations in unrelated individuals.(3) However, there are no large pedigrees with quantitative phenotype data for OI patients with the identical mutation.

The predominant type I collagen isotype in the extracellular matrix (ECM) is a heterotrimeric molecule comprised of two α1(I) chains and one α2(I) chain. A homotrimeric isotype of type I collagen, comprised of three α1(I) chains, is also found in tissues in small amounts in the nonpathologic state. The homotrimeric isotype is associated with autosomal recessive connective tissue disorders in humans when no α2(I) chains or only nonfunctional α2(I) chains are synthesized.(6,7) The amino acid sequences of both α1(I) and α2(I) chains are homologous and have a characteristic [Gly-X-Y]_338_ repeat within their respective triple-helical domains. The small side chains of glycine residues positioned at every third position of the repeat are sterically required for helix formation, and mutations in these glycine residues account for 80% of the known triple-helical mutations that cause moderately severe to lethal forms of OI.

Over the past two decades, several laboratories have developed murine OI models based on mutations in the proα1(I) chain of type I collagen patterned after those described in human OI to extend our understanding of OI pathobiology. All the existing mouse models have a moderate-to-severe bone phenotype with impaired viability. The two early models of note are the *Col1a1* null allele *Mov-13* mice(8–10) and the “protein suicide” or collagen minigene model.(11–13) The homozygous *Mov-13* mouse produces no type I collagen, and this is lethal. Heterozygous *Mov-13* mice deposit reduced amounts of normal type I collagen in the ECM that is associated with an osteopenic phenotype and abnormal skeletal biomechanics. The minigene model construct expresses a version of the human *COL1A1* gene that is missing the central 41 exons that encode a significant portion of the triple-helical domain. The shortened human proα1(I) chains associate with normal murine proα1(I) and proα2(I) chains, depleting the amount of normal type I collagen and altering ECM organization. Most recently, the *Cre*/*lox* recombination system was used to develop a *Col1a1* G349C substitution to reproduce a human OI type IV (moderately severe) phenotype termed *BrtlIV*.(14–17) The *BrtlIV* mouse represented a major advance in OI mouse models because it is the only *Col1a1* model with a triple-helical glycine mutation.

The OI phenotype pattern of proα2(I) glycine substitutions appears to be different from proα1(I) substitution.(3) Generally, proα2(I) mutations are less severe. They also may represent an easier target for treatment because only 50% of collagen molecules are affected in *COL1A2* heterozygotes versus 75% in *COL1A1* heterozygotes. However, the only reported mouse model for OI with a *Col1a2* mutation is osteogenesis imperfecta-murine (*oim*). Homozygous *oim* mice exhibit skeletal disease with clinical and biochemical features of Sillence type III (severe progressive) OI. The *oim* mutation and its biologic consequences(18–21) are strikingly similar to those found in a German child with type III OI.(6,22,23) In both instances, a frame-shift mutation in the region of the *COL1A2* gene that encodes the terminal portion of the proα2(I) C-propeptide predicts the synthesis of nonfunctional proα2(I) chains. Despite being the only naturally occurring OI mouse model, the *oim* mouse has a rarely observed type of mutation and an atypical biochemical phenotype because only proα1(I)_3_ homotrimeric molecules are formed.

The absence of a *COL1A2* murine OI model with autosomal dominant inheritance, mild to moderate disease, and phenotype variation represents a major gap in our tools to understand OI pathobiology and to develop effective treatment. Defining the molecular basis of phenotype variation also has been limited by the absence of large numbers of OI patients carrying the identical causative mutation with quantitative phenotype data to use as a prototype for a new OI mouse model. Here we report the clinical and biochemical phenotype of a *COL1A2* gene variant found among a Lancaster County, Pennsylvania, Old Order Amish (OOA) kindred. This *COL1A2* variant is a G-to-T transversion at nucleotide 2098 that alters the gly-610 codon (GGT) to a cysteine (TGT) codon.(24) We also report the creation of the first glycine-substitution knock-in *Col1a2* mouse that replicates the gene and protein phenotypes observed in the OOA kindred. The knock-in *G610C OI (Amish)* mouse represents a unique translational model for evaluating the molecular basis of phenotype variability and to test potential therapeutic strategies for OI.

## Materials and Methods

### Ethical considerations

All human subject research was reviewed and approved by the Institutional Review Board of the University of Maryland Baltimore (UMB). Informed consent was obtained for all study participants. All animal procedures were reviewed and approved by the UMB Institutional Animal Care and Use Committee.

### Recruitment of family members and phenotype assessment

The study had three stages of informed consent: (1) mutation screening, (2) clinical assessment, and (3) skin-biopsy harvest. The screening enrollment age requirement of 6 years or older was waived if one parent was mutation-positive by sequence analysis. After obtaining informed consent (or assent from the children), buccal cells were harvested as a source of genomic DNA (gDNA) for genotyping. All gDNA- and cDNA-derived sequence data (see below) were analyzed, using Sequencher software (GeneCodes, Ann Arbor, MI, USA).

All volunteers verified by gDNA sequence analysis to carry a single copy of the T allele (OI group) and their control (G allele) nuclear family members (age ≥ 6 years) were invited to participate in the clinical assessment and skin-biopsy phases. The phenotype assessment consisted of a medical and family history, physical examination, blood draws for serum procollagen type I C-terminal propeptide (PICP) and CrossLaps measurements (markers of bone formation and resorption), and measurement of areal bone mineral density (aBMD). Lumbar spine (L1–4) and proximal femur (total and subregions) aBMD was measured by dual-energy X-ray absorptiometry (DXA) on a QDR 4500W instrument (Hologic, Bedford, MA, USA), and the results reviewed by a single clinician (EAS) and another author (DJM). The in vivo coefficient of variation for replicate aBMD measurements was 0.8% for the lumbar spine, 0.9% for total hip, and 1.5% for the femoral neck. The Hologic Pediatric Reference Curve Option (hip: 5 to 20 years; spine: 3 to 20 years) and Caucasian reference (age > 20 years) were used to obtain age-specific *Z*-scores. Since OI is frequently associated with short stature and bone mineral content (BMC) and aBMD are influenced by bone size, volumetric bone mass [bone mineral apparent density (BMAD)] also was estimated to minimize the influence of small bone size.(25,26) A subset of the phenotype assessment volunteers also provided 3 mm dermal punch biopsies from the posterior upper arm from which fibroblasts were derived and stored at UMB and/or submitted to the Coriell Institute (OI families 1997, 1998, 2187, and 2229).

### Generation of knock-in mice

Knock-in mice were created using embryonic stem cells and *Cre*/*lox* P technology. A 13 kb genomic clone containing the relevant segment of the murine *Col1a2* gene was isolated from a λ phage library constructed with gDNA of the 129Sv/Ev Taconic mouse strain. PCR site-directed mutagenesis was used to change the targeted codon from GGT to TGT. The *Col1a2* G610C-positive embryonic stem cells were injected into blastocysts of C57BL/6J (B6) mice. Founder mice that retain the neo targeting vector are termed *neo+* or *G610C Neo mice*. Progeny obtained by breeding a founder male with a female that expressed Cre recombinase (Jackson Laboratory, Bar Harbor, ME, USA, Stock Number 003724) are termed *neo–* or G610C OI mice. The G610C OI mouse line is available to the research community through the Jackson Laboratory Mouse Repository (Jax Stock Number: 007248).

Four F_1_ strains of *G610C OI* mice were used to determine the effect of genetic background on phenotype in 2-month-old male mice. Incipient congenic *G610C OI* B6 (∼98% B6 genetic background) male breeders were generated by six generations of backcrosses to Jackson Laboratory B6 mice (Stock Number 000664). Experimental heterozygous B6 male breeders were crossed with A/J (Stock Number 000646), BALB/cByJ (Stock Number 001026), C3H/HeJ (Stock Number 000659), and FVB/NJ (Stock Number 001806) females purchased from the Jackson Laboratory. Progeny of these crosses are designated, respectively, as *A.B6*, *Cby.B6*, *C3.B6*, and *FVB.B6*. Experimental mice were housed at UMB in a single specific-pathogen-free room and were exposed to identical environmental conditions consisting of a 12 hour light/dark cycle, an ambient temperature of 23°C, and *ad libitum* access to water and laboratory mouse chow. Genotype was assigned using a PCR assay that can discriminate the three possible *G610C OI* mouse genotypes. The forward primer (TCC CTG CTT GCC CTA GTC CCA AAG ATC CTT) and the reverse primer (AAG GTA TAG ATC AGA CAG CTG GCA CAT CCA) will generate a 165 bp (wild type) or a 337 and a 165 bp (heterozygous) or a 337 bp (homozygous) PCR product using *G610C OI* mice gDNA. All animals were euthanized by CO_2_ asphyxiation.

### Human and mouse collagen analysis

Pepsin-soluble collagen was purified from mouse tail tendons and human fibroblast culture medium for analysis by SDS-PAGE and differential scanning calorimetry (DSC). Purified collagen samples were labeled by fluorescent monoreactive Cy5 NHS ester (GE Healthcare Bio-Sciences Corp., Piscataway, NJ, USA) or double-labeled by Cy5 and fluorescent BODIPY-L-Cys (Molecular Probes, Invitrogen, Inc., Carlsbad, CA, USA).(27) Labeled proteins were size-separated by SDS/PAGE on precast 3% to 8% gradient Tris-acetate minigels (Invitrogen). Following electrophoresis, the gels were briefly rinsed in distilled water and scanned on a Fuji FLA5000 fluorescence scanner (Fuji Medical Systems, Stamford, CT, USA) using a 473 nm excitation laser for BODIPY-L-Cys and a 635 nm laser for Cy5. For DSC, collagen was redissolved (1 to 2 mg/mL final collagen concentration) in 2 mM HCl (pH 2.7) or in 0.2 M sodium phosphate/0.5 M glycerol (pH 7.5) and then dialyzed extensively against the same buffer to remove excess salt. DSC thermograms(28) of approximately 0.1 mg/mL of pepsin-treated collagen in 2 mM HCl (pH 2.7) and in the phosphate/glycerol buffer (pH 7.5) were recorded in a Nano II differential scanning calorimeter (Calorimetry Sciences Corp. Lindon, UT, USA) at several different scanning rates. The apparent melting temperature *T*_m_ was defined at the maximum of the melting peak after baseline subtraction.

Total RNA was extracted from fibroblast cultures, and cDNA was synthesized using RACE1 [5′-GAT GGA TCC TGC AGA AGC (T)17-3′]. An aliquot of cDNA and forward exon primer and downstream reverse exon primer (selected to cross intron-exon) boundaries were used for second-strand synthesis and PCR amplification. PCR products of the expected size were confirmed by agarose gel size separation prior to purification for direct nucleotide sequencing.

### Phenotype assessment of F_1_ *G610C* OI Mice

#### aBMD measurements

The aBMDs of right femurs were measured by DXA (GE-Lunar densitometer, PIXImus; software version; 2.10, GE Medical Systems, WI, USA). Femur measurements were obtained by placing an excised femur on a Delrin block. The percent coefficient of variation (CV) was 1.3% for BMC and 1.4% for BMD.

#### Confocal Raman microspectroscopy

Segments of cortical bone from the femur diaphysis were embedded in Cryo-gel (Instrumedics, Inc., St. Louis, MO, OSA) and cut longitudinally normal to the bone surface on a Cryo-Cut One cryostat (Vibratome, Bannockburn, IL, USA). The 30 µm sections were examined with a Senterra confocal Raman microscope (Bruker Optics, Billerica, MA, USA). Raman spectra from 40 to 4500 cm^−1^ were collected with 9 to 18 cm^−1^ resolution using a circularly polarized 20 mW (9 mW at the sample), 532 nm laser beam, X40/0.95 NA objective, and a 50 µm confocal pinhole. The spectra were acquired at 12 points visually selected outside osteocytes along the periosteal to endosteal axis in the midplane of the sections. The accumulation time for each point was 1 minute. The mineral content of bone matrix (PO_4_:CH), collagen content of bone matrix (amide III:CH), and mineral:collagen ratio (PO_4_:amide III) were determined from the areas under ν_1_-PO_4_ (integrated from 901 to 987 cm^−1^), amide III (1214 to 1305 cm^−1^), and CH (2824 to 3035 cm^−1^) Raman peaks. Relative collagen content from Pro:CH ratio (833 to 867 cm^−1^) was consistent with that evaluated from the amide III:CH ratio but less accurate owing to low intensity of the Pro peak.

#### Femur µCT

Micro-computed tomography (µCT) was used to quantify femoral geometry, morphology, and mineralization (GE Medical Systems, London, ON, Canada). Femurs from F_1_ male 2-month-old mice were scanned over 200 degrees of rotation and reconstructed on 18 µm voxels. Cortical and trabecular analyses included measures of bone mineral content and density, cortical and trabecular thickness, and trabecular spacing. Cortical regions of interest (ROIs) consisted of cylindrical segments (radius 1.35 mm, height 3 mm) of the middiaphyses. Trabecular ROIs also consisted of cylindrical segments (radius 0.57 mm, height equal to 10% of total femur length) of the distal femoral metaphyses, proximal to the growth plates. Separate thresholds were applied to the cortical and trabecular ROIs (2000 and 1200 HU, respectively).

#### Ex vivo mechanical testing

Femurs were mechanically tested at room temperature using a four-point bending apparatus (858 Mini-Bionix, MTS, Eden Prairie, MN, USA) in a manner described previously by Jepsen.(10) With posterior femoral surfaces kept in tension, displacement was applied at 0.05 mm/s. The upper and lower tines of the four-point support were separated by 6.35 and 2.2 mm, respectively. Custom software was used to determine stiffness, yield, maximum load, and pre- and postyield fracture energy. Standard beam theory was used to calculate yield stress and Young's modulus based on the mechanical testing data and femoral geometry as measured by µCT.

#### Statistical analysis

Statistical analysis was performed using InStat and Prism statistical software packages (Graphpad, La Jolla, CA, USA) or SAS statistical software (SAS Institute, Cary, NC, USA). A two-tailed *p* value of .05 or less for any single analysis was considered statistically significant. Outliers (defined as values exceeding ± 2σ from the group mean) were removed from the µCT and four-point bending data sets.

## Results

### OOA kindred genotyping/pedigree structure

#### Putative founder couple identification

A 54-year-old woman with a history of fracture, low hip and spine aBMD, and a differential diagnosis of idiopathic osteoporosis or OI was identified in the Amish Family Osteoporosis Study (AFOS).(29,30) A brother and a niece of the woman also were identified with a history of fracture and low aBMD. gDNA was extracted from blood of the proband's brother for analysis of *COL1A1* and *COL1A2* genes.(31–33) Nucleotide sequencing identified a G-to-T substitution that converts the triple-helical codon for glycine-610 (GGT) to cysteine (TGT). Sequence analysis confirmed that the proband, brother, and niece carried a single copy of the mutant T allele. Three additional AFOS DNA samples (a grandmother, mother, and granddaughter) were found subsequently to carry single copies of the variant T allele.

**Fig. 1:**
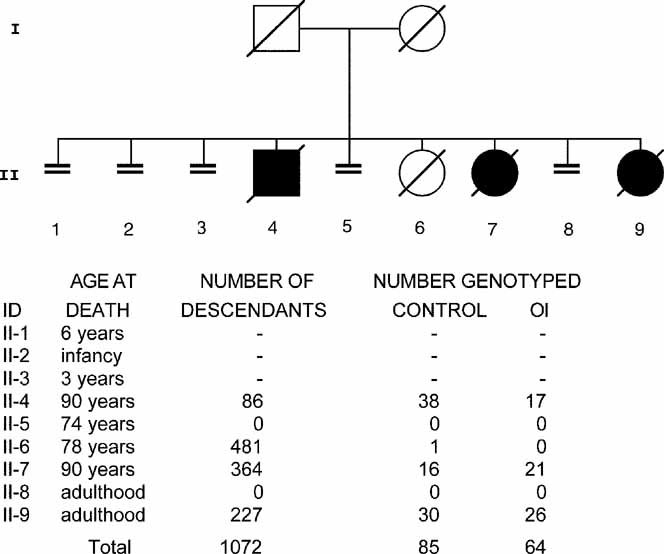
OOA *COL1A2* G610C putative founder couple pedigree. The *COL1A2* variant is a G-to-T transversion at nucleotide 2098 that alters the gly-610 codon (GGT) to a cysteine (TGT) codon. The mutant T allele has been mapped in 64 heterozygous descendants of the putative founder couple born approximately 150 years ago. Four of the founder couple's offspring have an estimated 800 living descendants, whereas the other five offspring produced no progeny. (Control genotype = GG; OI genotype = GT.)

The Anabaptist Genealogy Database (AGDB4)(34,35) was queried using PedHunter(36) software to link the six individuals with the mutant T allele to a putative founder couple (Fig. 1) born approximately 150 years ago. AGDB genealogic records listed approximately 1200 descendents from four of the putative founder couple's children, of which approximately 800 are estimated to be alive today. Genotype status was obtained for 195 study participants; 149 were direct descendants of the putative founder couple, 19 were direct-descendant married-in spouses, and 27 were descendants of the putative founder couple's siblings or more distant relatives. The variant T allele was identified in 64 descendants in 22 sibships ranging in age from 3 weeks to 86 years. Sibships ranged from 2 to 11 individuals with an average of approximately 6 individuals. The overall sibship genotype distribution exhibited a normal Mendelian 1:1 ratio expected for offspring from one carrier parent and one wild-type parent. No homozygous individuals and no carrier × carrier couples were identified.

### OOA kindred phenotype

#### Standing height

**Fig. 2:**
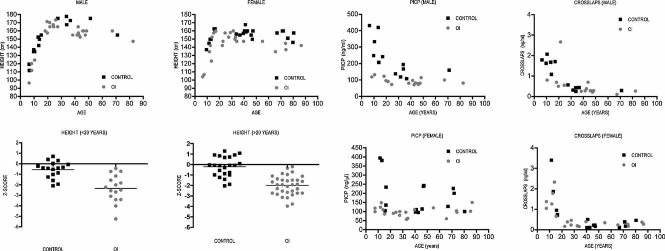
OOA kindred standing-height and serum biomarker measurements. Height (*left four panels*): Male (*left*) and female (*right*) carriers (GT) of the *COL1A2* G610C allele (OI) tend to have reduced standing height compared with control (GG) family members. Standing height *Z*-scores for individuals younger than 20 years of age were calculated using NHANES normative data, whereas an OOA-specific *Z*-score was calculated for individuals older than 20 years of age. The mean OI *Z*-score for standing height was significantly reduced (*p* < .0001) compared with family controls for both age groups. Biomarkers (*right four panels*): PICP (*left*) and CrossLaps (*right*) are serum biomarkers associated with collagen metabolism. PICP reflects collagen biosynthesis in bone. PICP values exhibited variations with age and disease status. The participants heterozygous for the G610C *COL1A2* allele (OI) who were younger than 20 years of age exhibited significantly reduced PCIP as compared with normal control family members. CrossLaps levels reflect the degradation of bone collagen and can be considered a surrogate for bone resorption. No obvious association with age or disease status was observed for the CrossLaps data.

Standing height as a function of age (Fig. 2) suggested that OI family members tended to be shorter than their unaffected family members. Standing height was converted to *Z*-scores to better assess the effect of the T allele across the wide age range of study participants. OOA-specific standing height *Z*-scores were generated separately for women and men from height data obtained from 1687 females and 1416 males (age > 20 years) enrolled in non-OI research studies (unpublished UMB data). Since OOA-specific data were unavailable for study participants 6 to 20 years of age, standing height *Z*-scores were generated using the National Health and Nutrition Examination Survey (NHAHES) data (https://web.emmes.com/study/ped/resources/htwtcalc.htm). Mean standing height *Z*-score was significantly less (*p* < .001) for carriers of the T allele as compared with control family members but with appreciable overlap in *Z*-scores between control and OI study participants (see Fig. 2).

#### Biomarker measurements

PICP values for OI family members were relatively constant as a function of age (see Fig. 2). However, PICP for control family members younger than 20 years of age were markedly higher compared with OI family members. Genotype (*p* < .0001) and age (*p* < .0086) were found to make significant contributions in a multiple regression model using age, sex, and genotype as variables for PICP. In contrast, only age (*p* < .0001) was found to make a significant contribution to serum CrossLaps.

#### BMD measurements

**Fig. 3:**
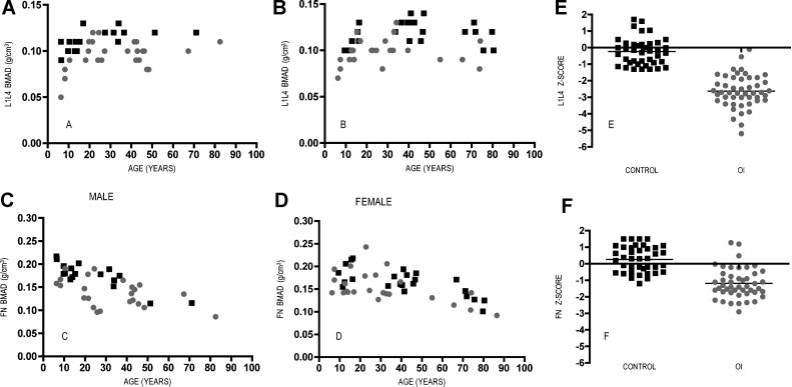
OOA kindred bone mineral density (DXA) measurements. The principal quantitative measure of phenotype severity was aBMD using DXA. BMAD was calculated from L1–4 spine (*A*, *B*) and femoral neck (*C*, *D*) DXA aBMD measurements as an estimate of volumetric bone density. Both male (*left*) and female (*center*) T allele individuals (OI) tended to have reduced BMAD compared with family controls as a function of age. Mean *Z*-scores using Hologic normative date are reduced in OI family members as compared with controls in both the spine (*E*) and hip (*F*).

Lumbar spine (L1–4) and femoral neck (FN) BMAD by age shown for males and females suggested that the OI group has reduced BMAD compared with family controls. Age, sex, genotype, and weight were used as predictor variables in multiple regression models for lumbar spine and FN BMAD. Sex (*p* < .03) and genotype (*p* < .0001) made significant contributions to lumbar spine and FN BMAD. Weight (*p* < .0001) also contributed to lumbar spine BMAD, and age (*p* < .0001) contributed to FN BMAD. BMD assessed as *Z*-scores for the spine and hip are shown in Fig. 3. Mean *Z*-score differences between the OI and control groups at the lumbar spine and the FN were significant (*p* < .0001). The mean *Z*-score at the lumbar spine was −2.63 ± 0.99 for the OI group and −0.24 ± 0.82 for the control group. Mean FN *Z*-scores were −1.18 ± 0.91 for the OI group and 0.26 ± 0.78 for the control group.

### Production of the knock-in mice

Nucleotide gDNA sequence (GenBank Accession Identifier DQ377843) analysis demonstrated that F_1_ founder mice (male = 1, female = 2) were heterozygous for the knock-in mutation in *Cola2* exon 33 (equivalent to *COL1A2* exon 35). Intron 32 for the F_1_ founder knock-in allele [*Col1a2*^*tm1Mcbr*^; Mouse Genome Informatics (MGI) ID 3805459] contains 2712 nucleotides, whereas the wild-type B6 allele contains 730 nucleotides. F_1_ founder mice cDNA sequence analysis confirmed expression of mutant and wild-type mRNA. Offspring carrying one or two copies of the knock-in *Col1a2*^*tm1Mcbr*^ allele from F_1_ founder breeding survived without any detectable lethality at birth or beyond weaning and were fertile.

Genomic DNA sequence analysis confirmed the *Cre*-mediated excision of the Floxed neo cassette in an F_2_ male mouse that was subsequently used to sire *G610C OI* mice progeny on a B6 background. Intron 33 in *G610C OI* mice is comprised of 852 nucleotides, and its MGI allele designation is *Col1a2*^*tm1.1Mcbr*^ (MGI Accession Identifier 3711122). It differs in size from the wild-type B6 sequence by the addition of 144 bp of residual targeting vector sequence that flanked the *lox* P sites and the loss of 22 bp of B6 sequence (GenBank Accession Identifier DQ377844). Heterozygous × wild-type breeding produced litters with both expected genotypes. However, no viable homozygous offspring were obtained from heterozygous matings. Genotype analysis of dead pups recovered within 24 hours of birth did demonstrate the presence of pups homozygous for the *Col1a2*^*tm1.1Mcbr*^ allele in the litters.

### Human and mouse SDS-PAGE analysis

**Fig. 4:**
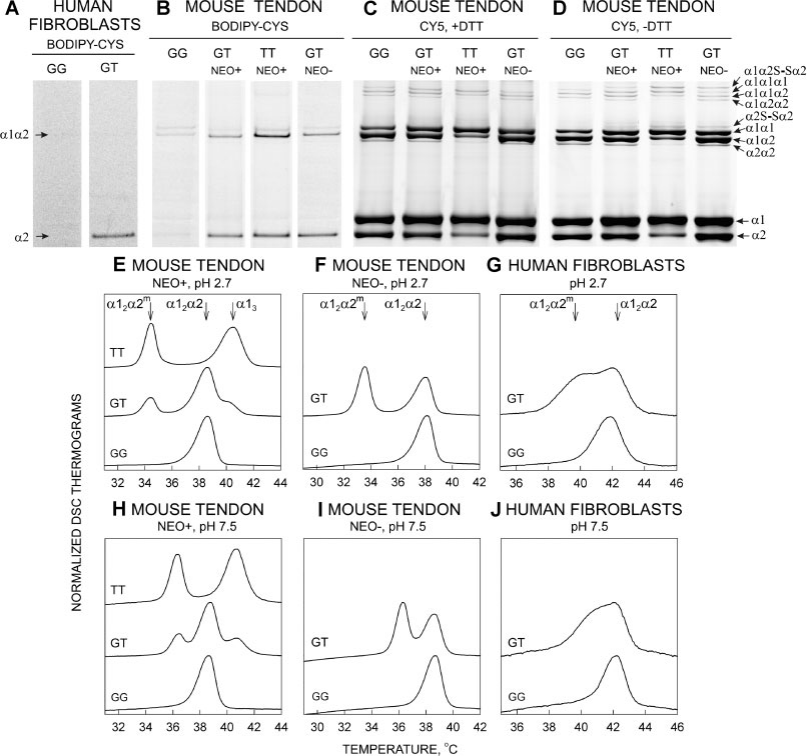
Type I collagen analysis. Type I collagen from human fibroblast cultures and from mouse tail tendons was analyzed by SDS-PAGE (*A–D*) and DSC (*E–J*). G610C genotype status is represented as GG (control), GT (heterozygous), and TT (homozygous). Neo+ represents the *Col1a2^tm1Mcbr^* allele in G610C mice, and neo– represents the *G610C OI* mouse *Col1a2^tm1.1Mcbr^* allele. The gels labeled by Cy5 and Bodipy-Cys are shown only in the Bodipy-Cys fluorescence (*A*, *B*). The Cy5 fluorescence of these gels was used for identification of the bands and correction for nonspecific Bodipy-Cys labeling (the residual nonspecific labeling still can be observed in the dimer bands). DTT was included in the sample loading buffer in panel (*C*) and excluded from the sample buffer in panels (*A*), (*B*), and (*D*). Normalized DSC thermograms of purified pepsin-treated collagen from *G610C* (neo+) and *G610C OI* (neo–) mouse tail tendons and human fibroblasts were measured at pH 2.7 (2 mM HCl; *E–G*) and pH 7.5 (measured in 0.2 M sodium phosphate and 0.5 M glycerol and corrected by 1.7°C to represent physiological conditions; *H–J*). Each peak represents the heat of denaturation of a distinct molecular form of type I collagen. Relative areas under the peaks on each thermogram represent the fraction of the corresponding molecules in the mixture. The superscript m identifies mutant α2(I) chains.

Double labeling with Cy5 and Bodipy-L-Cys revealed intense Cys staining of α2(I) chains from all mutant animals and proband collagens (Fig. 4A, B), indicating the presence of exposed reactive Cys-SH residues in α2(I) chains of type I collagen triple helices. Interestingly, SDS-PAGE also revealed the presence of reducible α2-Cys-S-S-Cys-α2 dimers in tendons of all mutant animals (see Fig. 4C, D). Since type I collagen triple helix contains only one α2 chain, such dimers must form between two different molecules. The intermolecular dimers likely form prior to fibrillogenesis because in the fibrils the Cys residues of adjacent triple helices are axially separated by at least one D period. Dimer formation likely occurs in the Golgi stack by nonspecific side-by-side interactions of procollagen molecules. Surprisingly, the aberrant intermolecular dimers are incorporated into the tissues of mutant animals. From the intensities of the bands containing the S-S dimers and corresponding bands without the dimers, the estimated fraction of mutant chains involved in the dimers was approximately 1% in heterozygous neo+, approximately 1.5% in heterozygous neo–, and approximately 9% in homozygous neo+ animals (Table 1).

**Table 1:** Differential Scanning Calorimetry (DSC) and SDS-PAGE Analysis of Type I Collagen

		DSC			SDS-PAGE		
			
Sample	Genotype	α1_2_α2, %	α1_2_α2^m^, %^a^	α1_3_, %	α1_3_, %	Reactive Cys-SH	α2^m^–S–S–α2^m^, %^a^
Mouse	GG	100	0	0	0	0	0
Mouse neo+	GT	59 ± 5^b^	17 ± 1^b^	24 ± 5^b^	23 ± 5	Yes	1 ± 0.3
Mouse neo+	TT	0	38 ± 1^b^	62 ± 1^b^	67 ± 5	Yes	9 ± 3
Mouse Neo–	GT	51 ± 1^b^	49 ± 1^b^	0	0	Yes	1.5 ± 0.3
Human	GG	100	0	0	0	No	—
Human	GT	50 ± 10^b^	50 ± 10^b^	0	0	Yes	—

a The superscript m identifies mutant chains.

b Estimated deconvolution error.

SDS-PAGE analysis also demonstrated an abnormally high ratio of α1(I):α2(I) fluorescence intensities in neo+ (*Col1a2*^*tm1Mcbr*^ allele) animals, approximately 3:1 in heterozygous neo+ and approximately 8:1 in homozygous neo+ versus approximately 2:1 in wild-type and heterozygous neo– (*Col1a2*^*tm1.1Mcbr*^ allele) animals. Most likely this was caused by insufficient synthesis of mutant α2(I) chains and formation of α1(I) homotrimers [e.g., owing to partial degradation and/or slower transcription, splicing, and/or translation of the neo+ (*Col1a2*^*tm1Mcbr*^) mRNA containing the intron 33 targeting vector]. Based on this assumption, we estimated the homotrimer content as approximately 23% and approximately 67% in tendons of heterozygous and homozygous neo+ animals correspondingly (see Table 1).

### Human and mouse DSC analysis

Previous differentail scanning calorimetry (DSC) studies showed that the denaturation temperature *T*_m_ of type I collagen homotrimers at acidic pH is approximately 2.5°C higher than that of normal heterotrimers.(28,37) Thus, to test whether the high α1(I):α2(I) ratio was consistent with homotrimer formation, we performed DSC scans of collagen from all mouse tendons and human fibroblast cultures in 2 mM HCl (pH 2.7). In addition to the denaturation peak of normal type I heterotrimers, the DSC thermograms of collagen from neo+ animals (see Fig. 4E) did reveal a low-temperature peak consistent with mutant heterotrimers and a high-temperature peak. The high-temperature peak was identical to *oim* and artificial homotrimers measured at the same conditions.(28,38) This peak was absent in collagen from neo– animals (see Fig. 4F) and from human proband fibroblasts (see Fig. 4G). Deconvolution of the DSC thermograms suggested approximately 24% and approximately 62% content of homotrimers in heterozygous and homozygous neo+ animals, respectively, in agreement with SDS-PAGE.

To evaluate the effect of the G610C substitution on the thermal stability of mutant collagen, we measured similar DSC thermograms in 0.2 M sodium phosphate and 0.5 M glycerol (pH 7.5), which can be used to evaluate the *T*_m_ under physiologic conditions by subtraction of 1.7°C. From the buffer-corrected thermograms (see Fig. 4H–J), we estimate that the incorporation of the mutant α2(I) chain results in a *T*_m_ reduction by 2.3 °C in mouse and approximately 1°C in human collagens correspondingly.

### F_1_ *G610C OI* mouse phenotype

#### Breeding

A total of 301 experimental mice were obtained from 48 litters. Genotype was determined for the mice, except for one B6.Cby female mouse. Mean litter sizes differed among the four F_1_ stains (one-way ANOVA *p* = .0039). The mean litter sizes were 7.9 ± 1.9 (FVB.B6), 6.5 ± 2.5 (Cby.B6), 5.0 ± 2.2 (C3.B6), and 5.0 ± 1.5 (A.B6). Goodness-of-fit testing indicated a significant deviation from expected genotype ratios looking at data from all litters (*p* = .008). However, no individual strains had a significant deviation from expected ratios.

#### Body weight

**Fig. 5:**
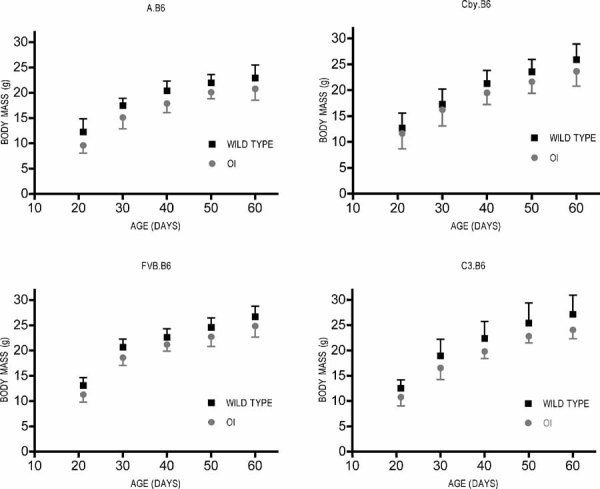
F_1_ *G610C OI* mouse growth curves. Body-weight curves demonstrated a curvilinear relation between weight and age for all groups of mice. Strain and genotype were independent predictors of weight. The wild-type (control; *black squares*) mice were 1.77 g heavier than the OI mice (heterozygous for the *Col1a2^tm1.1Mcbr^* allele; *gray diamonds*), with the greatest difference in body weight between the FVB.B6 and the A.B6 groups.

There was a curvilinear relation between body weight (measured longitudinally on days 21 to 60) and age (Fig. 5; age and age squared both *p* < .0001). Wild-type animals were heavier than OI animals (difference 1.77 g, SE 0.3279, *p* < .0001). The A.B6 mice had the lowest and the FVB.B6 mice had the greatest body weight. FVB.B6 mice were significantly heavier than A.B6 mice (difference 2.07 g, *p* < .0001), FVB.B6 mice were significantly heavier than Cby.B6 mive (difference 1.37 g, *p* < .003 (both *p* values adjusted for multiple comparisons using Tukey-Kramer). There was no evidence of a strain-by-genotype interaction (strain × genotype *p* < .8).

### aBMD of *G610C OI* mouse femurs

*G610C OI* mice had lower BMC (Δ = 0.005 g, *p* < .001) and BMD (Δ = 0.005 g/cm^2^, *p* = .001). Mean aBMD rank order of the *G610C OI* mice by maternal background strain for was A.B6 < Cby.B6 < FVB.B6 < C3.B6. Pairwise comparisons of the *G610C OI* mice using the Scheffe multiple-comparisons adjustment procedure resulted in a statistically significantly difference between A.B6 OI and C3.B6 OI (Δ = 0.007 g/cm^2^, *p* = .0061). A strain-by-genotype interaction was not significant for either BMC or BMD (*p* ≈ .9).

### Confocal Raman microspectroscopy

**Fig. 6:**
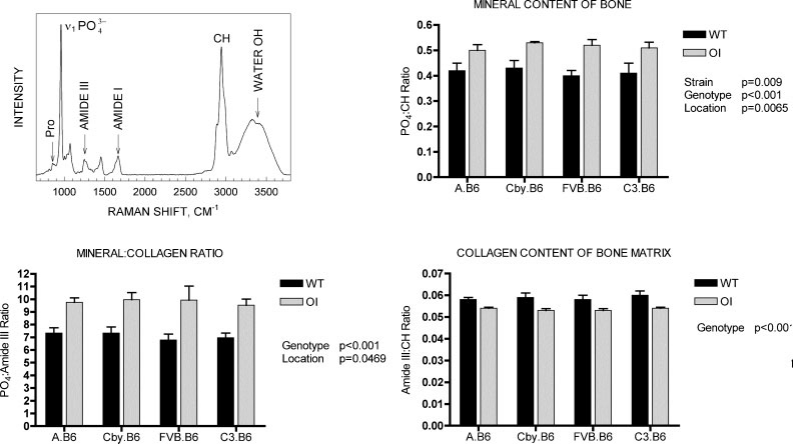
F_1_ *G610C OI* mouse Raman microspectroscopy. The collagen content of the OI (heterozygous for the *Col1a2^tm1.1Mcbr^* allele) femurs was reduced relative to wild-type (WT) control femurs, and the collagen was hypermineralized. A representative Raman spectrum illustrates the peaks used for data analysis (upper *left*), and comparison of chemical-specific ratios in WT (*black bars*) and *G610C OI* (*gray bars*) femurs determined from the Raman spectra are shown in the balance of the figure.

Mineral content of bone matrix (PO_4_:CH) (Fig. 6) was found to be affected by genotype (*p* < .0001) and mouse strain (*p* = .009). A significant curvilinear relationship was observed between mineral content and bone location (*p* = .0065). The mineral:collagen ratio (PO_4_:amide III) also was found to have a significant genotype (*p* < .0001) effect and a curvilinear relationship between mineral content and location (*p* = .047) but no evidence of a strain effect (*p* = .36). The curvilinear relationships for PO_4_:CH and PO_4_:amide III had peak values located around the middle of the cortical bone. Only genotype was found to have a significant (*p* < .0001) effect for the collagen content of bone matrix (amide III:CH). Mouse strain-by-genotype interaction was not significant for any of the Raman data.

### µCT

Table 2 shows mean values of selected trabecular and cortical bone parameters obtained from isolated femora. Cortical shape and femur length were qualitatively very similar for each background strain control-OI pair (data not shown). Overall, the µCT trabecular and cortical data show that all *G610C OI* mice carrying one copy of the *Col1a2*^*tm1.1Mcbr*^ allele had less bone as measured by bone volume fraction. The trabecular bone was distinguished by fewer and more widely spaced trabeculae. Except for the FVB.B6 mice, the mean cortical thickness and area were reduced by the presence of the *Col1a2*^*tm1.1Mcbr*^ allele. The FVB.B6 control and OI groups had similar mean values for cortical thickness and area. All the OI femurs had reduced moment of inertia (*I*_*yy*_) compared with age-matched genetic background controls. The cortical volumetric tissue mineral content (vTMC) was lower in all OI mice relative to their genetic background controls, except for the FVB.B6 OI group, which was the same as the FVB.B6 control group. The cortical volumetric tissue mineral density (vTMD) was elevated in all mice carrying *Col1a2*^*tm1.1Mcbr*^ allele relative to their genetic background controls. The trabecular vTMD was statistically the same for each genetic background pair.

**Table 2:** Trabecular and Cortical Parameters Derived From µCT

	A.B6		Cby.B6		FVB.B6		C3.B6		ANCOVA *p* value		
					
Mean SD	WT	OI	WT	OI	WT	OI	WT	OI	Interaction	Genotype	Strain
Trabecular BV/TV (%)	0.082	0.059	0.200	0.106	0.148	0.083	0.172	0.091		<.0001	<.0001
	0.026	0.026	0.076	0.046	0.020	0.014	0.068	0.026			
Trabecular number	2.923	1.798	4.793	2.845	4.501	2.561	4.051	2.247		<.0001	<.0001
	0.781	0.491	1.114	0.859	0.434	0.473	1.197	0.713			
Trabecular vBMD	469.400	488.500	526.000	513.700	459.900	473.200	533.300	534.700			<.0001
	10.860	35.650	33.330	41.980	7.096	22.020	18.550	38.690			
Cortical bone vol. fraction (%)	0.12	0.10	0.15	0.12	0.14	0.13	0.16	0.13	.0309		
	0.0076	0.003	0.019	0.014	0.0083	0.011	0.016	0.014			
Cortical thickness (mm)	0.18	0.17	0.20	0.18	0.18	0.19	0.23	0.20	.0094		
	0.0084	0.0076	0.019	0.016	0.0067	0.011	0.015	0.014			
Cortical area (mm^2^)	0.66	0.58	0.81	0.68	0.76	0.74	0.904	0.72	.0154		
	0.044	0.018	0.102	0.079	0.047	0.061	0.086	0.077			
Cortical I_yy_ (mm^4^)	0.082	0.061	0.13	0.086	0.12	0.103	0.13	0.083		<.0001	<.0001
	0.0090	0.0049	0.027	0.016	0.014	0.015	0.022	0.021			
Cortical vTMC (mg)	2.00	1.80	2.55	2.13	2.28	2.26	2.84	2.29	.0124		
	0.14	0.07	0.38	0.28	0.15	0.20	0.29	0.30			
Cortical vTMD (mg/mL)	999.48	1031.56	1019.20	1041.7	969.32	1025.33	1048.21	1056.83	.0052		
	9.51	13.20	22.83	21.07	13.26	22.46	14.21	26.27			

### Four-point bending biomechanics

Isolated femurs were loaded to failure in a four-point bend-testing apparatus to assess the impact of a single copy of the *Col1a2*^*tm1.1Mcbr*^ allele and the role of genetic background on the structural phenotype (Table 3). Specifically, femurs from mice with one copy of the variant allele were significantly weaker and more brittle than femurs from control mice. In addition, a genotype-by-genetic-background (strain) interaction was significant for failure load, stiffness, postyield ultimate displacement, and energy to failure. The presence of the *Col1a2*^*tm1.1Mcbr*^ allele resulted in a striking reduction in failure load for all F_1_ OI mice compared with their strain-specific controls. The mean failure load range was greater for control mice (24 N for A.B6 mice to 35 N for C3.B6) compared with OI mice (15 N for A.B6 mice to 18 N for C3.B6 mice). The impact of the *Col1a2*^*tm1.1Mcbr*^ allele on the magnitude of the intrastrain control-OI difference in stiffness was variable. The greatest intrastrain OI-control mean difference was found in the C3.B6 group, where the *Col1a2*^*tm1.1Mcbr*^ allele was associated with a 71% reduction in mean stiffness. The OI mice in the other three groups had mean stiffness values of 82% (A.B6), 91% (Cby.B6), and 98% (FVb.B6) of their intrastrain controls. Mean values for postyield ultimate displacement for OI mice were 45% (Cby.B6), 52% (C3.B6), and 59% (FVB.B6) of strain-matched control values. However, the mean value for A.B6 OI mice was 95% of the control value. Failure energy ranged from 0.78 (Cby.B6) to 1.14 N-mm (C3.B6) for the OI mice and from 3.11 (A.B6) to 6.3 N-mm (FVB.B6) for controls.

**Table 3:** Structural and Predicted Material Parameters Derived From Four-Point Bending

	A.B6		Cby.B6		FVB.B6		C3.B6		ANCOVA *p* value		
					
Mean SD	WT	OI	WT	OI	WT	OI	WT	OI	Interaction	Genotype	Strain
Failure load (N)	24.24	14.86	28.76	15.20	27.38	17.82	34.51	17.99	.0481		
	1.92	2.68	6.02	2.35	3.89	2.024	2.78	4.24			
Stiffness (N/mm)	187.48	154.51	190.78	174.06	194.81	190.74	256.68	181.75	.0204		
	26.11	13.85	37.66	28.092	28.18	27.069	26.92	27.05			
Postyield displacement (mm)	0.0038	0.0036	0.0058	0.0026	0.0063	0.0037	0.0063	0.0033	.0430		
	0.00053	0.00013	0.00018	0.00016	0.00010	0.00013	0.00011	0.00015			
Ultimate stress (MPa)	425.00	334.04	415.82	276.80	377.12	283.47	437.00	354.13		<.001	.0094
	53.42	61.37	40.79	47.44	30.10	33.97	10.81	95.81			
Young's modulus	8.025	9.53	6.124	7.75	5.88	7.30	7.39	9.30		.0001	.0003
	1.63	1.24	1.14	1.04	0.99	1.32	0.87	2.63			
Yield energy	0.69	0.67	1.44	0.602	0.72	0.76	1.25	0.83	.0002		
	0.17	0.19	0.50	0.13	0.086	0.045	0.33	0.31			
Failure energy (N-mm)	3.11	0.88	5.66	0.78	6.30	1.03	5.31	1.14	.0002		
	0.56	0.35	1.24	0.25	1.41	0.32	1.34	0.55			

Ultimate stress and Young's modulus had statistically significant genotype and strain differences, but the genotype-by-genetic-background interaction was not significant (see Table 3). Mean ultimate stress was reduced for all OI groups relative to their genetic background controls and ranged from 67% (Cby.B6 OI mice) to 81% (C3.B6 OI mice). Young's modulus was increased across all OI groups carrying the *Col1a2*^*tm1.1Mcbr*^ allele relative to their genetic background controls. The magnitude of the mean Young's modulus was not different between OI-control pairs but varied by genetic background of the strain.

## Discussion

The ascertainment of 64 related individuals harboring the mutant T allele represents the largest reported collection of OI patients with an identical collagen mutation. Amino acid substitutions at invariant glycine residues typically result in clinically apparent phenotypes and make ascertainment more likely. While the original brother-sister-niece proband cluster presented with some of the most severe clinical signs and symptoms of OI in this kindred, our overall clinical data support a Sillence type I (mild) to type IV (moderate) phenotype range. Pedigree analysis suggests that the T allele was a de novo mutation that occurred in the putative founder couple born circa 1860. The rapid OOA population growth from approximately 600 individuals in 1860 to about 31,000 children and adults today,(39) combined with stochastic events, endogamy, and large sibships, likely contributed to the mutation's expansion within the Lancaster Settlement.

Fracture history is the most relevant phenotypic trait used to make a clinical diagnosis of OI and to classify disease severity. However, since radiographic and medical record documentation of fractures were unavailable in the OOA kindred, the use of fracture history was problematic in defining the range of phenotypic severity associated with the T allele. The self-reported fracture number (not shown), nevertheless, indicated that the OI group was more prone to fracture and reinforced a clinical diagnosis of OI. Instead, DXA measurements of aBMD were used as a continuous quantitative trait to assess phenotypic severity that reflected the Sillence OI type clinical spectrum. Prior work suggests that mean aBMD for children and adults with OI types III and IV is significantly reduced compared with controls, but a significant number of OI type I patients had aBMD values within the reference range.(40–42) Non-OOA OI patients often have L1–4 spine *Z*-scores in the −1 to −2 range for type I OI and the −2 to −4 range for type IV OI patients (JCM clinical experience). These stratification ranges demonstrate that the T allele was associated with a wide range of disease severity (Table 4) in the spine and a milder phenotype in the hip.

**Table 4:** DXA *Z*-Score Stratification

		Spine (L1–4)		Femoral neck (FN)	
			
Sillence classification^a^	Z-score range	Control (GG)	OI (GT)	Control (GG)	OI (GT)
Normal	> −1	31	2	39	16
Type I	−1 to −2	9	11	1	22
Type IV	−2 to −4	0	32	0	8
Type III–IV	< −4	0	3	0	0
Total number		40	48	40	46

a Stratification by Sillence type is based on clinical experience (JCM) with non-OOA OI patients,

Skeletal growth deficiency is a cardinal secondary feature of OI that is reflected in the standing-height measurement. Type I OI patients typically attain a subnormal to normal adult height, whereas type IV OI patients, with a greater propensity for fragility fracture, have a shorter final standing height in the range of pubertal children.(43) Figure 2 demonstrates a consistent reduction in standing height of T allele carriers compared with kindred controls (mean *Z*-score difference of approximately 1.8 in both the <20- and >20-year-old groups). During childhood and adolescence, some 90% of skeletal mass is attained,(44) and peak stature normally is attained by approximately 20 years of age. The cross-sectional serum biomarker data suggest impaired skeletal accretion and linear bone growth in T allele carriers; PICP levels were relatively flat across the entire age spectrum for T allele carriers (see Fig. 3). In normal, healthy children, PICP levels reflect collagen biosynthesis associated with skeletal growth and are known to change significantly with age and pubertal development stages.(45–47) In contrast to the PICP estimates of bone formation, bone resorption (serum CrossLaps) had similar age and sex patterns for both the OI and control groups. This suggests an uncoupling between bone formation and resorption for the OI group during a major period of bone mass accretion that favors a net decrease in bone mass.

The OOA kindred and the *G610C OI* mouse share the classic OI features of dominant inheritance and a triple-helical glycine substitution. The biomedical significance of this kindred is its clinical and quantitative prototypic varied disease severity that is further enhanced by its rich resource of phenotype data, DNA, and fibroblast cell lines. However, repeated access to kindred bone samples and other tissues for experimental uses are unlikely, thus necessitating creation of the *G610C OI* mouse to aid future studies. Establishing the validity of the *G610C OI* mice as the first α2(I) glycine substitution model required the following elements: (1) duplication of the codon change, (2) expression of mutant proα2(I) transcript, (3) deposition of mutant protein in the ECM, and (4) confirmation of a bone phenotype.

Our initial evaluation of founder (neo+) mice and their progeny carrying the *Col1a2*^*tm1Mcbr*^ allele demonstrated that the G610 codon was altered as planned and the mutant transcript was expressed. Surprisingly, these mice secreted a significant fraction of type I collagen homotrimer in addition to G610C-containing heterotrimeric collagen. The formation of homotrimers appeared related to intron 32 targeting vector retention because it was not detected in human patients and neo– mice. Therefore, *G610C OI* (or neo–) mice carrying the *Col1a2*^*tm1.1Mcbr*^ allele actually model the human condition accurately at the gene, transcript, and protein levels. However, the neo+ animals are of interest and important to understand because G610C (neo+) mice carrying both one and two copies of the *Col1a2*^*tm1Mcbr*^ allele were viable yet had a very mild phenotype by torsional testing of femurs (data not shown). This altered phenotype associated with production of homotrimers suggests that some OI patients with α2(I) mutations may benefit from treatments designed to shift the balance of the chain synthesis to favor homotrimer production.

Given that phenotype variation was a significant clinical feature observed among the OOA kindred, the impact of the genetic background on phenotype severity was tested in *G610C OI* mice carrying the *Col1a2*^*tm1.1Mcbr*^ allele. Heterozygous × wild-type mouse crosses resulted in good reproductive fitness that matched the reproductive fitness of the OOA kindred and yielded F_1_ strains with approximately 50% interstrain genetic background identity. Interestingly, reproductive fitness of heterozygous × heterozygous B6 matings was poor because trial matings failed to produce offspring homozygous for the *Col1a2*^*tm1.1Mcbr*^ allele that survived beyond the perinatal period.

Growth curves (body weight) from weaning to 2 months of age demonstrated impaired weight gain for all four F_1_ mouse strains carrying the *Col1a2*^*tm1.1Mcbr*^ allele relative to their controls. This is likely a generalizable effect in mice caused by type I collagen gene mutations because it has been reported for the *Mov13*, *oim*, and *BrtlIV* OI models. Body weight is a complex quantitative trait,(48) and different inbred mice are exemplified by characteristic patterns of weight gain with time (MGI Database). It is likely that the different growth curves reflect the maternal contribution to the genetic background of the F_1_ mice because inbred mouse strains exhibit characteristic patterns of weight gain with age.

**Fig. 7:**
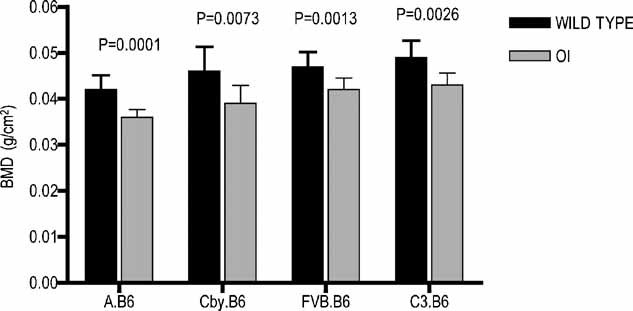
F_1_ *G610C OI* mouse DXA. OI mice demonstrated a reduction in mean whole-femoral BMC and aBMD relative to their genetic background wild-type (WT) controls. The rank order of the OI (heterozygous for the *Col1a2^tm1.1Mcbr^* allele) mice by background strain for aBMD means was A.B6 < Cby.B6 < FVB.B6J < C3.B6.

Since the degree and nature of bone matrix mineralization may be an important determinant of resistance to fracture, whole-femur mineral mass in F_1_ mice was assessed by aBMD and µCT measurements. PIXImus measurements (Fig. 7) demonstrated that all strains of mice carrying the *Col1a2*^*tm1.1Mcbr*^ allele had a reduction in aBMD compared with controls, and this is consistent with DXA data from the OOA kindred. However, µCT measurement of increased cortical vTMD (see Table 3) better reflects the tissue effects of the variant *Col1a2* allele. This apparent contradiction is explained by the 2D nature of aBMD estimated by DXA versus the 3D data obtained by µCT. The µCT data suggest the *G610C OI* mice have reduced total tissue mineral content but also have reduced total bone mass that results in greater bone mineralization compared with control mice. Hypermineraliztion at the level of collagen fibers was confirmed by Raman microspectroscopy and is consistent with higher mean mineralization density in OI patient bone samples measured by backscattered electron imaging.(49)

Structural integrity of femoral cortical bone was assessed using four-point bending, which is an ex vivo surrogate in mice for increased long bone fracture risk associated with OI. The structural (failure load) and material property (ultimate stress) measures of femur strength at 2 months of age demonstrated that a single copy of the *Col1a2*^*tm1.1Mcbr*^ allele reduced mechanical integrity regardless of genetic background. Key contributors to the structural soundness of bone include geometric parameters (i.e., size, shape) and inherent material properties such as the molecular integrity, quantity and organization of type I collagen, and degree and nature of collagen mineralization. While the mineral content of bone contributes substantially to its stiffness, collagen tends to dominate the properties of the postyield region of the load-deformation curve. It would appear from the yield energy:total energy ratio that once the bone starts to break, the presence of the mutated collagen offers less resistance to complete failure in *G610C OI* mice. The failure of the abnormal collagen heterotimer to “toughen” the bone is likely due to a structural and/or organizational defect caused by the mutation and/or a general reduction in the amount of collagen per unit volume of bone. Our human and mouse findings of reduced DSC denaturation temperature *T*_m_ suggest that the global molecular (structural) stability of the mutant collagen heterotrimer is weakened. This reduction in *T*_m_ is commonly associated with OI collagen mutations, although there is no simple relationship between the magnitude of *T*_m_ decrease and OI disease severity. Reduced bone matrix collagen content (measured by Raman microspectroscopy) also appears to contribute to loss of femoral structural integrity in *G610C OI* mice. The variations in the murine bone biomechanical properties across the several experimental strains used in this study (genotype-by-strain interactions) provide additional insight about skeletal complications among human patients. The effect of the mutation on the estimated bone tissue properties was the same across all strains, each demonstrating significant and similar reductions in inherent tissue integrity. However, the magnitude of structural property alterations (such as failure load) appears to be dependent on genetic background (strain). Taken together, these results suggest that the effect of the mutation on basic, inherent bone tissue integrity is similar in all OOA kindred patients (reduced modulus and increased brittleness). However, the consequence of the defect on whole-bone fracture susceptibility is influenced by individual genomic factors that are reflected in size, shape, and possibly bone metabolic regulation.

Mouse models have the advantage over human samples of tight control of genetic background and the ability to circumvent or minimize the role of environment on phenotype. The F_1_ *G610C OI* screening crosses were successful in demonstrating that genetic background variation affected bone geometry and structural property phenotype. We speculate that additional backcrosses will further magnify interstrain phenotype differences and enhance the probability of identifying major genetic modifiers using standard and novel backcross and intercross genetic mapping tools with proven success in the detection of human disease gene modifiers.(48,50) Bone trait modifiers identified in the *G610C OI* mouse likely will include *Col1a1* or *Col1a2* mutation-specific modifiers, some that are generalizable for many OI mutation loci, and others that overlap with genes associated with common forms of osteoporosis. Quantitative trait locus (QTL) investigation in the *G610C OI* mouse also likely will confirm QTLs found in prior osteoporosis studies, thus narrowing the critical QTL regions.(51,52) The OOA OI kindred and AFOS data then can be used to confirm both sets of genes—those common to osteoporosis and those unique to Ol.

In summary, the OOA kindred is the largest known OI kindred with an identical type I collagen defect and extensive phenotype data. The triple-helical glycine substitution and dominant inheritance pattern are prototypic for most reported cases of OI. While all the affected individuals share the same mutation, they varied widely in phenotype, as quantified by aBMD *Z*-scores. The *G610C OI* mouse, patterned on the OOA family, duplicates its gene and protein profile. The magnitude of structural property alterations owing to the mutation was dependent on genetic background (strain). The bone geometry and structural property gene × genetic background interactions suggest that noncollagen genes can modify the *G610C OI* mouse OI phenotype. Overall, these results demonstrate that *G610C OI* mice represent a unique translational OI model to define the complex genetic architecture controlling phenotype and for testing of potential therapeutic approaches and treatment strategies for OI.
